# Comparing measurement‐derived (3DVH) and machine log file‐derived dose reconstruction methods for VMAT QA in patient geometries

**DOI:** 10.1120/jacmp.v15i4.4645

**Published:** 2014-07-08

**Authors:** Neelam Tyagi, Kai Yang, Di Yan

**Affiliations:** ^1^ Department of Medical Physics Memorial Sloan‐Kettering Cancer Center New York NY USA; ^2^ Department of Radiation Oncology William Beaumont Hospital Troy MI USA

**Keywords:** 3DVH, machine log file, dose reconstruction

## Abstract

The purpose of this study was to compare the measurement‐derived (3DVH) dose reconstruction method with machine log file‐derived dose reconstruction method in patient geometries for VMAT delivery. A total of ten patient plans were selected from a regular fractionation plan to complex SBRT plans. Treatment sites in the lung and abdomen were chosen to explore the effects of tissue heterogeneity on the respective dose reconstruction algorithms. Single‐ and multiple‐arc VMAT plans were generated to achieve the desired target objectives. Delivered plan in the patient geometry was reconstructed by using ArcCHECK Planned Dose Perturbation (ACPDP) within 3DVH software, and by converting the machine log file to Pinnacle^3^ 9.0 treatment plan format and recalculating dose with CVSP algorithm. In addition, delivered gantry angles between machine log file and 3DVH 4D measurement were also compared to evaluate the accuracy of the virtual inclinometer within the 3DVH. Measured ion chamber and 3DVH‐derived isocenter dose agreed with planned dose within 0.4%±1.2% and ‐1.0%±1.6%, respectively. 3D gamma analysis showed greater than 98% between log files and 3DVH reconstructed dose. Machine log file reconstructed doses and TPS dose agreed to within 2% in PTV and OARs over the entire treatment. 3DVH reconstructed dose showed an average maximum dose difference of 3% ± 1.2% in PTV, and an average mean difference of ‐4.5%±10.5% in OAR doses. The average virtual inclinometer error (VIE) was ‐0.65° ± 1.6° for all patients, with a maximum error of ‐5.16° ± 4.54° for an SRS case. The time averaged VIE was within 1°–2°, and did not have a large impact on the overall accuracy of the estimated patient dose from ACPDP algorithm. In this study, we have compared two independent dose reconstruction methods for VMAT QA. Both methods are capable of taking into account the measurement and delivery parameter discrepancy, and display the delivered dose in CT patient geometry rather than the phantom geometry. The dose discrepancy can be evaluated in terms of DVH of the structures and provides a more intuitive understanding of the dosimetric impact of the delivery errors on the target and normal structure dose.

PACS number: 87.55

## I. INTRODUCTION

Our routine pretreatment quality assurance (QA) approach involves performing a QA measurement in a homogeneous water‐equivalent phantom and comparing it to the planning system calculations derived in a similar water‐equivalent phantom geometry. However, the effect on real planning structures cannot be quantified when performed and analyzed in a phantom geometry. The need to quantify doses to patient anatomy is becoming more essential for complex VMAT deliveries. The simultaneous movement of MLCs, gantry, and variability of dose rate may potentially be more prone to delivery errors than conventional planning and may have a bigger dosimetric impact due to these delivery errors. Current IMRT QA devices, such as film, ion chamber, and diode and ion chamber arrays, can be used for VMAT delivery QA; however, ion chamber and films are limited in measuring doses to a point or 2D plane. Previous studies have reported on the use of an electronic portal imaging device (EPID) for reconstructing delivered doses^(1)^ or the use of machine log files for reconstructing planned fluence and dose in patient anatomy.^(2)^ Recently ArcCHECK device, from Sun Nuclear Corporation, has been analyzed for homogeneous water‐like patient geometries by Nelms et al.^(3)^ for VMAT deliveries and by Olch^(4)^ for IMRT deliveries. These studies were, however, limited to homogeneous geometries. More recently, ArcCHECK has been validated in heterogeneous phantom geometries. Lin et al.^(5)^ performed an ArcCHECK comparison in an inhomogeneous geometry consisting of ArcCHECK phantom without the cavity insert and compared the exit dose measurements with Monte Carlo simulation, AAA based TPS dose calculation, and MatriXX ion chamber dosimeter. The authors found more than 98% gamma agreement between MC and ArcCHECK, and 97% between TPS and ArcCHECK when 1 mm dose grid size was used for dose calculation. Both the entrance and exit doses measured by ArcCHECK, with and without the cavity plug, agreed within 1% when compared with Monte Carlo simulation. The agreement between ArcCHECK without the cavity plug and TPS was 7% when a 2.5 mm dose calculation grid size was used. But the agreement improved significantly with a smaller calculation grid size.

At our institution, we modified the existing ArcCHECK geometry by designing a lung insert in the central cavity of the detector, and dosimetrically monitored with MOSFETS in the air cavity and compared the delivered dose with the calculation.^(6)^ This was achieved by the use of a custom made water‐equivalent sphere with five embedded MOSFETS. This sphere was encapsulated in a lung insert that can be accommodated by both the cylindrical QA phantom and a CIRS lung phantom. ArcCHECK absolute dose analysis between measurement and calculation using gamma analysis (3%/3 mm) showed more than 98% of diodes passing for both static and dynamic phantom with and without the lung phantom insert. MOSFET static measurement showed 2% agreement with the calculated value (5450±120 vs. 5250±20 cGy).

Recently 3DVH software has been developed by Sun Nuclear Corporation that converts the measurements taken in a cylindrical acrylic geometry into actual patient CT geometries, using a measurement derived perturbation algorithm (also called ArcCHECK Planned Dose Perturbation or ACPDP). 3DVH takes into account all the potential delivery errors that have been propagated during the measurements. The final results can then be analyzed in terms of DVHs of various plan structures and represent the dose delivered to the patient in the CT anatomy taking into account all potential measurement and delivery uncertainty. Opp et al.^(7)^ performed a comparison between 3DVH and a CIRS lung phantom with OSL inserts to validate the dose to the heterogeneity using four TG‐119 recommended VMAT plans. In less modulated plans, the agreement was 100%; however, the agreement dropped to 96% for complex shapes. The authors contribute this effect to the limitation of TPS. However, this needs to be validated using a fully benchmarked Monte Carlo‐based TPS. In this study, we compare the 3DVH dose reconstruction method with our in‐house‐developed dose reconstruction method derived from machine log files.^(8)^ Our dose reconstruction method has been thoroughly verified in a previous publication, and has shown excellent agreement with the planned doses for the target.^(8)^

The purpose of this study is to compare and benchmark the accuracy of two methods against each other. Both methods are capable of taking into account the plan delivery errors. The difference in analysis using these two methods will be primarily due to sensitivity of ArcCHECK on plan delivery errors, measurement uncertainty related to ArcCHECK device, and the difference in dose reconstruction algorithms between the two methods. This study is of significance because many institutions rely on machine log files for their patient‐specific QA.^9^, ^10^ Schreibman et al.^(2)^ have also used machine log file‐based methodology for patient specific QA for VMAT delivery. If analyzed properly, log files will tell us if the MLC leaf sequences entered at the workstation are delivered with sufficient clinical accuracy. The log files analysis will also report delivered dose errors due to dropped segments, leaf over/under shoot, faulty leaf motors, tongue‐and‐grove effect, rounded leaf ends, and communication delays between the controller and the workstation. In addition, they also tell us if the MLC gain calibration is set appropriately.^8^, ^11^

## II. MATERIALS AND METHODS

### A. ACPDP algorithm

Absolute dose distribution for the delivered plan was measured using the acrylic ArcCHECK cylindrical diode array (Sun Nuclear Corporation, Melbourne, FL) and 3DVH version 2.2. The technical details of 3DVH software analysis and ACPDP algorithm have been described in detail by Nelms et al.^(4)^ We describe a few main points for completeness. The 3DVH is a QA tool that estimates the dose distribution in the patient without the patient present, using input from conventional phantom QA methods. Time‐resolved ArcCHECK measurement, gantry angle determined by virtual inclinometer, and DICOM RT plan are synchronized to create a modulated fluence per subbeam in phantom ($∼2^{\circ }$ intervals). This time‐resolved subbeam fluence is then convolved with a pre‐optimized 3D pencil beam kernel, and transformed into absolute composite dose in phantom using entrance and exit diode's absolute measured dose, as well as a global correction factor, described in detail by Nelms et al.^(4)^ The estimated delivered patient dose comes from a voxel‐by‐voxel correction of the TPS planned dose on patient, according the dose differences between TPS and ACPDP on phantom.

Before using the 3DVH software, a linear accelerator model library needs to be set up to enable the ACPDP algorithm. If treatment machines are missing from the library, the users will be prompted to add the machines when loading data. ArcCHECK measurement is saved as a text file and a movie file (*.acml or *.acm) that contains the calculated gantry angles for each measurement, in addition to the 4D measured data. The setup geometry consists of the ArcCHECK device filled with the cavity plug made of acrylic material. In addition, a custom‐made insert for a Farmer style ion chamber was also placed at the center of the cavity plug to measure the isocenter dose using the ion chamber. Charge collected was converted to absolute dose by applying a calibration factor from a 10 × 10 cm^2^ measured dose. 3DVH software also gives an estimate of the measured ion chamber dose at the center and gives a range of doses within a 1 cc volume.

### B. Treatment plans

VMAT treatment plans were generated using Pinnacle's SmartArc algorithm (Philips Healthcare, Andover, MA) for various treatment sites, and fractionation schedule as shown in Table 1. A total of ten patient plans was selected from a series of a regular fractionation plan to complex SBRT plans. Treatment sites in the lung and abdomen were chosen to explore the effects of tissue heterogeneity on the respective dose reconstruction algorithms. Spine radiosurgery cases were chosen because the steep dose gradient between the target and adjacent critical normal structures will be sensitive to treatment delivery errors.

All the SBRT cases were simulated using 4D CT scans, and the plans were generated using the average CT set obtained from the 4D CT, per our institution policy. Single and multiple arcs were generated for these plans to achieve the desired target objectives.^(12)^ The table describes a summary of prescription, dose fractionation, total monitor units, and VMAT QA results. Phantom isocenter dose from the planned dose (from TPS) and measured dose (from ion chamber and 3DVH), along with the differences with respect to the planned dose, is also shown. Absolute dose measured using ArcCHECK for all cases are also shown in Table 1. All plan calculations and QA calculations were interpolated to 2° control point spacing to mimic the actual VMAT delivery, and calculated with a $2\times 2\times 2\;{\text{mm}}^{3}$ dose grid size. These plans were generated for a 6 MV Elekta machine equipped with a beam modulator using 4 mm MLC leaves. A gamma analysis criteria of 3% absolute dose error and 2 mm distance to agreement (DTA) was used to compare the measured and calculated dose.^(13)^ Detectors receiving at least 4% of the prescription dose were included in the analysis. In addition to QA analysis in cylindrical geometries, QA measurement was reconstructed in patient CT geometry using the 3DVH software tool.

**Table 1:** Summary of prescription and VMAT QA results for all treatment sites. Multiple entries for Patient 10 represent three lesions treated using a single isocenter

					*Phantom Isocenter Dose (Gy)*				
*Treatment Site*	*Pat*	*Dose Fx*	*# of Arcs*	*Total MU*	*TPS*	*IC*	$\%diff_{IC}$	*3DVH*	$\%diff_{3DVH}$
Prostate	1	1.8Gy/fx×37	1	353.1	70.4	71.3	1.3	69.9	−0.7
SBRT Prostate	2	3.2Gy/fx×20	1	644.4	73.5	73.9	0.5	73.8	0.4
SBRT Lung	3	12Gy/fx×5	1	2550.0	59.5	60.7	2.0	58.3	−2.0
	4	12Gy/fx×4	1	2177.9	52.0	53.0	−1.9	52.4	0.8
	5	12Gy/fx×4	2	1349.1	54.8	55.4	1.5	53.9	−1.6
				1349.9					
Rib	6	2.5Gy/fx×16	1	391.0	30.6	30.5	−0.3	30.6	0.1
SBRT Liver	7	20Gy/fx×3	1	4446.3	62.7	63.5	1.3	61.7	−1.6
SRS Spine	8	18Gy/fxx1	2	1683.0	16.9	16.8	0.6	16.9	0.2
				3665.3					
SBRT Spine	9	7Gy/fx×5	2	901.3	37.6	37.3	−0.8	37.5	−0.3
				1038.7					
SRS Ischial	10	8Gy/fx×1	1	1251.3	8.1	8.1	−0.5	7.7	−4.9
SRS Femoral		8Gy/fx×1	1	609.4					
SRS Symphysis		8Gy/fx×1	1	1461.0					

$\%{\text{diff}}_{\text{IC}}=(\text{IC}-\text{IPS})/\text{TPS}$; $\%{\text{diff}}_{3\text{DVH}}=(3\text{DVH}-\text{IPS})/\text{TPS}$.

### C. Dose reconstruction using machine log files

A previously developed dose reconstruction tool was used in this study.^(5)^ The tool is written in C++ and converts the machine log file output from VMAT deliveries into Pinnacle^3^ 9.0 treatment plan format. The reconstructed plans were imported to Pinnacle and recalculated to obtain the actual delivered dose in patient anatomy. The reconstructed doses were compared with planned dose and 3DVH reconstructed doses in terms of changes in target and normal structure doses. In addition to planned and reconstructed doses, delivered gantry angles between machine log files and 3DVH 4D files were also compared for all cases to evaluate the accuracy of the virtual inclinometer within the 3DVH software tool.

## III. RESULTS

### A. Target dose: PTV minimum, maximum, and mean dose

Target isocenter doses was compared between the planned value and measured value from ion chamber and 3DVH, as shown in Table 1. Charge from ion chamber was converted into dose by applying a $10\times 10\;{\text{cm}}^{2}$ calibration measurement obtained at the same time. ${\text{Diff}}_{\text{IC}}$ and ${\text{Diff}}_{3\text{DVH}}$ are differences in isocenter dose (in Gy) between ion chamber and planned dose and 3DVH and planned dose, respectively. The measured dose values are shown as total prescribed dose rather than dose per fraction. The dose was measured per fraction and then scaled by the number of fractions to obtain total dose. Measured ion chamber doses and 3DVH‐derived isocenter doses agree within 2% with the planned dose for all cases investigated in this study. Single fraction plan shows bigger discrepancy between 3DVH reconstruction and planned dose, whereas the ion chamber dose at the isocenter agrees very well.

Table 2 shows the 3D gamma dose comparison between various methods. The AC vs. TPS is the ArcCEHCK QA measurement vs. the original pretreatment plan calculation. The AC vs. logfile is the ArcCHECK QA measurement vs. the log file reconstructed plan calculation. The percent difference between the first and second column shows that the reconstructed plan can better represent the delivery by considering the delivery error as compared to the pretreatment plan. The last column represents the 3D gamma dose comparison between log file reconstructed dose and 3DVH reconstructed dose, and compares the delivered dose in patient using the two methods. The 3D gamma comparisons for all cases are 98% or higher, which shows a very good agreement between the two methods.

**Figure 1:**
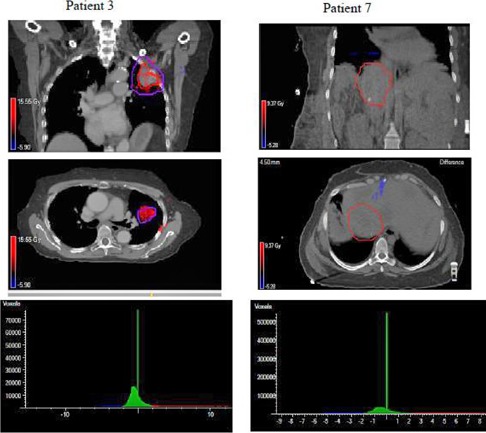
Coronal and axial slice showing hot & cold region from a dose difference comparison for Patient 3 and Patient 7, respectively, along with the histogram distributions of planned vs. delivered doses.

**Figure 2:**
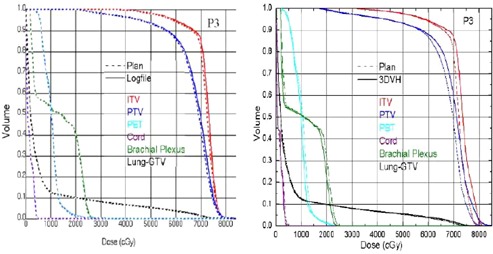
DVH comparison showing the ${\text{diff}}_{\text{lo}}$ (left) and the ${\text{diff}}_{3\text{DVHd}}$ (right) for a SBRT lung case (Patient 3) ${\text{iff}}_{\log}=(\text{logfile}-\text{TPS});{\text{diff}}_{3\text{DVHd}}=(3{\text{DVH}}_{\text{deliver}}-3{\text{DVH}}_{\text{plan}})$.

Tables 3, 4, and 5 show the minimum $\left(D_{99.9}\right)$, maximum, and mean doses from the treatment plan (within the TPS and as imported in 3DVH), as well as the measured deviations as reconstructed by the machine log files and 3DVH. Plan 3DVH describes the reference plan loaded in 3DVH software. We found differences in plan references values for minimum, maximum, and mean dose values as compared to Pinnacle TPS, when the reference plan was loaded into 3DVH. This discrepancy is mainly due to the way voxels are binned into 3DVH for different organs. Based on our study, we saw differences of up to −21.4% (or −1.2 Gy) in minimum dose, 2.8% (or 1.9 Gy) in maximum dose, and less than 1.3% in mean doses in just the reference plan dose (labeled as ${\text{diff}}_{3\text{DVHp}}$). We believe minimum dose (reported as $D_{99.9\%}$ from 3DVH and Pinnacle TPS) is very sensitive to any interpolation or binning performed internally in loading the CT and dose matrix. For consistency sake, 3DVH reconstructed plans were compared with the reference plan loaded in 3DVH and machine log file‐reconstructed plans were compared with the reference plan in the Pinnacle TPS. Table 2 also shows the absolute dose difference in Gy between doses reconstructed from machine log file and plan file (labeled as ${\text{diff}}_{\log}$) and dose reconstructed using 3DVH and plan data imported into 3DVH (labeled as ${\text{diff}}_{3\text{DVHd}}$). 3DVH in general showed bigger discrepancy in reconstructed dose and planed dose as compared to that from machine log file dose and planed dose. Dose difference of 3.8% (or 3.2 Gy) and 4.8% (0.8 Gy) in maximum doses (Table 4) for the target was observed in Patient 3 (lung SBRT case) and Patient 8 (SRS spine), respectively. Mean PTV doses were within 2 Gy between 3DVH reconstructed and plan dose. Differences of up to 4.7% (or 0.4 Gy) in mean dose was seen for single fraction cases. Both heterogeneity and location of tumor contributed to the observed large discrepancy. For Patient 3, the tumor was located at the periphery of lung. ACPDP applies a perturbation based on the ratio of the ACPDP measured dose and the ArcCHECK planned dose for each voxel to the TPS patient dose for that same voxel. An estimated patient dose for that voxel is thus calculated based on this perturbation, which also includes the inhomogeneity accuracy of the TPS, just like the machine log file calculation. Since the inhomogeneity correction maybe similar in both methods, larger systematic errors in estimated delivery dose in 3DVH compared to machine log files could be due to the limitations in the ratio approach used in the 3DVH to estimate patient dose. Figure 1 shows the hot and cold regions for Patients 3 and 7 in coronal and axial planes, along with the histogram distribution for the dose comparison between 3DVH reconstructed dose and plan dose. Hot region in Patient 3 target area represents the systematic overestimated impact of the delivery error on estimated delivered dose from 3DVH in all there SBRT lung cases. Line shaped cold region in Patient 7 shows the evidence of beam modeling difference in modeling the transmission between 3DVH and Pinnacle. Figure 2 shows a DVH comparison of the planned dose and estimated delivery dose reconstructed using log file and 3DVH for Patient 3, which showed the largest dose discrepancy in both the target and OARs between two methods.

**Table 2:** 3D gamma comparison analysis for all treatment sites. AC vs. TPS represents ArcCHECK QA comparison with the original pretreatment plan; AC vs. log file represents the ArcCHECK QA comparison with the log file reconstructed plan calculation; log file vs. 3DVH represents the log file reconstructed plan comparison with the 3DVH reconstructed dose

	*3D Gamma Analysis (3%/2 mm)*			
	*ArcCHECK*			*Patient*
*Pat*	*AC vs. TPS*	*AC vs. Logfile*	*Diff*	*Logfile vs. 3DVH*
1	96.0	96.8	0.8	99.3
2	90.7	92.9	1.2	98.3
3	92.6	91.8	−0.8	99.2
4	97.7	98.1	0.4	99
5	92.0	91.8	‐0.2	99.4
	92.3	91.6	‐0.7	
6	96.0	95.9	‐0.1	99.7
7	86.4	93.7	7.3	97.9
8	98.2	98.5	0.3	98.1
	95.1	96.5	1.4	
9	93.8	93.8	0.0	99.7
	93.7	94.0	0.3	
10	95.9	96.9	1.0	99.2
	98.9	98.9	0.0	
	98.7	99.6	0.9	

**Table 3:** Minimum PTV dose comparison between planned dose (TPS & 3DVH) and delivered doses reconstructed from machine log file and 3DVH. Planned 3DVH dose corresponds to reference plan imported into 3DVH

	*Minimum PTV Dose* ^(a)^ *(Gy)*						
	*Plan*			*Deliver*			
*Pat*	*TPS*	*3DVH*	$\%diff_{3DVHp}$	*Logfile*	$\%diff_{log}$	*3DVH*	$\%diff_{3DVHd}$
1	63.7	63.3	−0.6	64.5	1.3	64.4	1.7
2	58.6	60.1	0.3	58.9	0.5	62.5	4.0
3	20.2	18.2	−9.9	20.8	3.0	17.0	−5.9
4	36.0	30.2	−16.1	36.2	0.6	29.1	−3.6
5	45.8	46.2	0.9	46.2	0.9	48.0	3.9
6	33.5	35.2	5.1	33.6	0.3	35.2	0.1
7	53.5	55.4	3.6	53.9	0.7	55.7	0.5
8	3.3	3.7	12.1	3.4	3.0	4.0	8.1
	5.6	4.4	−21.4	5.8	3.6	4.2	−4.5
9	17.0	17.3	1.8	17.3	1.8	17.7	2.3
10	7.7	7.9	2.6	7.7	0.5	8.3	5.1
	7.8	7.9	1.3	7.8	0.5	8.3	5.1
	7.5	7.7	2.7	7.5	0.5	8.0	3.9

a $D_{99.9\%}$ is used for minimum dose.

$\%{\text{diff}}_{3\text{DVHp}}=(3{\text{DVH}}_{\text{plan}}-\text{TPS})/\text{TPS};\%{\text{diff}}_{\log}=(\text{logfile}-\text{TPS})/\text{TPS};\%{\text{diff}}_{3\text{DVHd}}=(3{\text{DVH}}_{\text{deliver}}-3{\text{DVH}}_{\text{plan}})/3{\text{DVH}}_{\text{plan}}$.

**Table 4:** Maximum PTV dose comparison between planned dose (TPS & 3DVH) and delivered doses reconstructed from machine log file and 3DVH. Planned 3DVH dose corresponds to reference plan imported into 3DVH

	*Maximum PTV Dose (Gy)*						
	*Plan*			*Deliver*			
*Pat*	*TPS*	*3DVH*	$\%diff_{3DVHp}$	*Logfile*	$\%diff_{log}$	*3DVH*	$\%diff_{3DVHd}$
1	72.4	72.8	0.6	73.2	1.1	73.8	1.4
2	67.8	68.7	1.3	68.4	0.9	69.8	1.6
3	82.3	84.5	2.7	83.7	1.7	87.7	3.8
4	65.7	66.3	0.9	66.0	0.5	68.2	2.9
5	60.7	60.8	0.2	60.9	0.3	62.2	2.3
6	44.2	44.7	1.1	45.1	2.0	45.6	2.0
7	67.1	69.0	2.8	68.1	1.5	69.9	1.3
8	10.6	10.7	0.9	10.6	0.4	11.0	2.8
	16.4	16.8	2.4	16.5	0.6	17.6	4.8
9	48.2	48.5	0.6	48.8	1.2	50.3	3.7
10	9.3	9.3	0.4	9.4	1.1	9.7	4.3
	9.3	9.3	0.4	9.3	0.4	9.7	4.3
	9.2	9.3	0.4	9.3	1.1	9.6	3.2

$\%{\text{diff}}_{3\text{DVHp}}=(3{\text{DVH}}_{\text{plan}}-\text{TPS})/\text{TPS};\%{\text{diff}}_{\log}=(\text{logfile}-\text{TPS})/\text{TPS};\%{\text{diff}}_{3\text{DVHd}}=(3{\text{DVH}}_{\text{deliver}}-3{\text{DVH}}_{\text{plan}})/3{\text{DVH}}_{\text{plan}}$.

**Table 5:** Mean PTV dose comparison between planned dose (TPS & 3DVH) and delivered doses reconstructed from machine log file and 3DVH. Planned 3DVH dose corresponds to reference plan imported into 3DVH

	*Maximum PTV Dose (Gy)*						
	*Plan*			*Deliver*			
*Pat*	*TPS*	*3DVH*	$\%diff_{3DVHp}$	*Logfile*	$\%diff_{log}$	*3DVH*	$\%diff_{3DVHd}$
1	68.6	68.6	0.1	69.4	1.2	69.3	1.0
2	66.0	66.4	0.6	66.5	0.8	66.9	0.8
3	66.4	66.7	0.5	67.0	0.9	68.3	2.4
4	58.1	58.8	1.2	58.4	0.5	60.4	2.7
5	53.7	53.9	0.4	54.0	0.6	55.0	2.0
6	41.9	42.3	1.0	42.4	1.2	42.6	0.7
7	62.9	63.2	0.5	63.6	1.1	63.6	0.6
8	7.8	7.9	1.3	7.8	0.5	8.1	2.5
	12.5	12.6	0.8	12.6	0.8	12.8	1.6
9	38.1	38.1	0.1	38.5	1.0	38.2	0.3
10	8.7	8.8	1.1	8.8	1.1	9.1	3.4
	8.7	8.8	1.1	8.8	1.1	9.1	3.4
	8.6	8.6	0.5	8.7	1.2	9.0	4.7

$\%{\text{diff}}_{3\text{DVHp}}=(3{\text{DVH}}_{\text{plan}}-\text{TPS})/\text{TPS};\%{\text{diff}}_{\log}=(\text{logfile}-\text{TPS})/\text{TPS};\%{\text{diff}}_{3\text{DVHd}}=(3{\text{DVH}}_{\text{deliver}}-3{\text{DVH}}_{\text{plan}})/3{\text{DVH}}_{\text{plan}}$.

### B. OAR doses: mean and maximum dose

Table 6 shows the comparison between planned doses and delivered doses (from machine log file and 3DVH) for the organs at risk (OAR). ${\text{Diff}}_{3\text{DVHp}},{\text{diff}}_{\log}$, and ${\text{diff}}_{3\text{DVHd}}$ have similar meanings as before. Mean and maximum doses for the parallel and serial organs were evaluated for the OAR discrepancy. Machine log file reconstructed doses and TPS dose agree to within 1‐1.5 Gy over the entire treatment course. This difference corresponds to up to 3% for some single fraction cases. 3DVH reconstructed doses are also within 1 Gy for majority of normal structures. However, the percent dose difference corresponds to up to 21% in maximum cord dose for single fraction cases. Cord represents the biggest maximum dose discrepancy in 3DVH analysis due to binning effect in the 3DVH system.

**Table 6:** Various OAR dose comparisons between planned dose (TPS & 3DVH) and delivered doses reconstructed from machine log file and 3DVH. Planned 3DVH dose corresponds to reference plan imported into 3DVH

		*Mean or Maximum Dose (Gy)*						
		*Plan*			*Deliver*			
*Pat*	*OAR1*	*TPS*	*3DVH*	$\%diff_{3DVHp}$	*Logfile*	$\%diff_{log}$	*3DVH*	$\%diff_{3DVHd}$
1	Rectum	71.2	71.4	0.3	72.2	1.4	73.8	3.4
2	Rectum	67.8	68.4	0.9	68.2	−0.6	69.7	1.9
3	PBT	31.1	26.0	−16.4	32.0	2.9	23.8	−8.5
4	Lung	4.7	4.7	0.9	4.8	2.1	4.7	0.9
5	Lung	5.3	5.4	1.9	5.4	1.9	5.4	0.7
6	Lung	6.7	6.7	1.5	6.8	1.5	6.6	−1.5
7	Cord	15.0	14.9	−0.7	15.3	2.0	14.3	−4.0
8	Cord	3.0	1.6	−46.7	3.1	3.3	1.7	6.3
		5.8	3.4	−41.4	5.9	1.7	2.7	−20.6
9	Cord	13.8	9.2	−33.3	14.1	2.2	8.4	−8.7
10	Bladder	8.5	8.4	1.2	8.6	1.2	8.7	3.6
		*Mean or Maximum Dose (Gy)*						
		*Plan*			*Deliver*			
*Pat*	*OAR2*	*TPS*	*3DVH*	$\%diff_{3DVHp}$	*Logfile*	$\%diff_{log}$	*3DVH*	$\%diff_{3DVHd}$
1	Bladder	70.6	70.6	0.1	71.7	1.6	71.7	1.6
2	Bladder	65.9	66.4	0.8	66.3	0.6	67.0	0.9
3	Cord	5.7	5.0	−12.3	5.8	1.8	4.5	−10.0
4	Esophagus	10.4	10.4	0.4	10.5	1.0	9.9	−4.8
5	PBT	28.8	27.0	−6.3	29.0	0.7	27.8	3.0
6	Liver	4.4	4.5	2.3	4.5	2.3	4.4	−2.2
7	Liver	22.7	26.0	14.5	23.0	1.3	25.8	−0.8
8	Parotid	2.3	2.3	1.7	2.3	1.7	2.3	1.7
		2.8	2.9	3.6	2.8	1.4	2.8	−3.4
9	Esophagus	16.9	16.6	1.8	17.0	0.6	16.6	1.4
10	Rectum	5.4	5.2	3.7	5.5	1.9	5.3	1.9
		*Mean or Maximum Dose (Gy)*						
		*Plan*			*Deliver*			
*Pat*	*OAR3*	*TPS*	*3DVH*	$\%diff_{3DVHp}$	*Logfile*	$\%diff_{log}$	*3DVH*	$\%diff_{3DVHd}$
1	Femoral Head	56.9	56.3	−1.1	57.3	0.7	57.5	2.1
2	Small Bowel	3.1	3.2	3.2	3.2	3.2	2.2	−31.3
3	Brachial Plexus	27.6	25.6	−7.2	29.1	5.4	24.0	−6.3
4	Brachial Plexus	26.3	25.7	−2.3	26.4	0.4	24.7	−3.9
5	Brachial Plexus	26.9	26.4	−1.9	27.1	0.7	26.6	0.8
6	Cord	2.3	2.5	8.7	2.3	1.7	2.5	1.6
7	Heart	22.6	23.4	−3.5	22.8	0.9	22.6	−3.4
8	Oropharynx	7.2	6.1	−15.3	7.1	−1.4	6.2	1.6
		6.5	6.3	−3.1	6.6	1.5	6.2	−1.6

$\%{\text{diff}}_{3\text{DVHp}}=(3{\text{DVH}}_{\text{plan}}-\text{TPS})/\text{TPS};\%{\text{diff}}_{\log}=(\text{logfile}-\text{TPS})/\text{TPS};\%{\text{diff}}_{3\text{DVHd}}=(3{\text{DVH}}_{\text{deliver}}-3{\text{DVH}}_{\text{plan}})/3{\text{DVH}}_{\text{plan}}$.

### C. Gantry angle comparison

**Figure 3:**
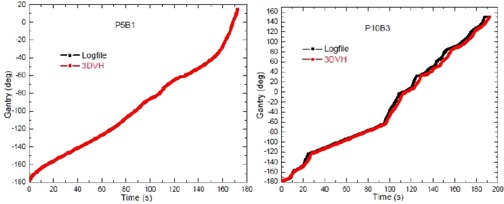
Gantry position as a function of time for a hypolung patient (Patient 5, beam 1) and a SRS patient (Patient 10, beam 3). Gantry position recorded in 3DVH and log file matches better in Patient 5 than in Patient 4.

Figure 3 shows the gantry angle comparison between machine log files and 3DVH measurement for two example patients. Two example cases are shown here that represents the minimum and maximum differences in gantry angles between machine log file and 3DVH measurements. Table 7 describes the gantry error in VMAT delivery as recorded in the machine log file, virtual inclinometer error (VIE), and integral virtual inclinometer error (IVIE), each calculated for ArcCHECK measurement as:

$$\begin{array}{rl} & Virtual\;Inclinometer\;Error\;(VIE)=C\;(Gantry_{3DVH}-Gantry_{log})\\ & Integral\;Virtual\;Inclinometer\;Error\;(IVIE)=C\int (Gantry_{3DVH}-Gantry_{log})dt+Delivery\;Time\\ & C=1\;or\;-\;1\;for\;CW\;or\;CC\;gantry\;rotatation \end{array}$$

The average VIE is $-0.65^{\circ }\pm 1.6^{\circ }$, and varies up to $-5.16^{\circ }\pm 4.54^{\circ }$ among all the patients. Considering dose delivery in VMAT is a continuous process, the time averaged VIE is introduced and defined as IVIE. It thus serves as a quantitative measure of the virtual inclinometer accuracy in VMAT delivery. Constant (either 1 or ‐1) is introduced to make the sign meaningful, so that positive or negative VIE (IVIE) means the timestamp recorded in 3DVH for a certain gantry angle is either earlier or later than that in log file. In general, the IVIE is within 1°–2°. It is not expected to have a large impact on the overall accuracy of the estimated patient dose from ACPDP algorithm, which is reconstructed based on subbeam fluences reconstructed in a 2° interval. However, up to 5° IVIE was seen for Patient 10.

The discrepancy in Patient 10 arises from a series of sudden gantry acceleration deceleration process. Since 3DVH has an inherent inclinometer, the real‐time gantry angles are calculated based on the entrance and exit photons hitting the phantom. An error can be expected in the inherent inclinometer calculation whenever subbeam path changes suddenly.

**Table 7:** Gantry angle comparison (also called the gantry error) between machine log file and 4D ArcCHECK measured movie file. The accuracy of the virtual inclinometer was quantified by calculating the integral virtual inclinometer error.(b)

*Treatment Site*	*Pat*	*Delivery Time (s)*	*Gantry Error (deg)*	*Virtual Inclinometer Error* ^a^ *(deg)*	*Integral Virtual Inclinometer Error* ^b^ *(deg)*
Prostate	1	115.5	0.22±0.5	−2.18±2.45	−2.59
Hypo Prostate	2	118.2	−0.06±0.5	−1.00±4.33	−1.53
Hypolung	3	353.5	−0.08±0.1	−1.00±1.5	−1.24
	4	282.3	0.03±0.1	−0.53±0.84	−0.60
	5	172.6	0.07±0.2	−0.13±0.38	−0.27
		175.9	−0.08±0.2	−0.01±0.50	−0.55
Rib	6	78.0	0.17±0.3	1.28±1.06	1.04
Hypoliver	7	581.8	0.01±0.1	−0.52±0.90	−0.58
SRS Spine	8	223.9	0.03±0.1	2.17±1.93	2.04
		481.3	0.00±0.1	−0.86±1.30	−0.80
Hypospine	9	121.2	0.09±0.3	−0.34±0.49	−0.56
		138.5	0.03±0.1	−0.18±0.36	−0.29
SRS Ischial	10	167.6	−0.09±0.2	−1.12±1.08	−1.23
SRS Femoral	92.7	0.08±0.4	−0.50±0.85	−0.81	
SRS Symphysis	192.3	−0.04±0.6	−5.16±4.54	−5.13	

a *Virtual Inclinometer Error* $\left(VIE)=C(Gantry_{3DVH}-Gantry_{\log}\right)$

*Integral Virtual Inclinometer Error* $(IVIE)=C\int (Gantry_{3DVH}-Gantry_{\log})dt÷Delivery\;Time$

C=1 *or* −1 *for CW or CC gantry rotatation*

b Positive or negative VIE (IVIE) means the timestamp recorded in the 4D movie file for the corresponding gantry angle is earlier or later than that in logfile.

## IV. DISCUSSION

In this study we have compared two independent dose reconstruction methods. Dose reconstructed from machine log files agrees more closely to the planned dose distribution as the dose calculation algorithm used to recalculate doses from the reconstructed fluence is the same. 3D gamma analysis between 3DVH and machine log file reconstructed dose showed greater than 98% agreement for all the cases investigated; however, bigger differences were seen for both target and normal structures when dose reconstructed from 3DVH was compared with the planned dose. A major contribution to the larger discrepancy is coming from the import of the planned dose in 3DVH itself. We saw big discrepancy in just comparing the planned doses between Pinnacle and the original plans loaded into 3DVH. The previous investigators did not observe this discrepancy, and is a matter of concern when using this software for routine QA purpose. Homogeneous patient geometries (such as prostate cases) agree well with data by Nelms et al.^(3)^ Heterogeneous geometries showed bigger difference than homogeneous geometries. 3DVH uses preloaded kernel libraries for various linear accelerators and some of the discrepancies can be expected just from the stored kernels. In addition to preloaded kernels, 3DVH determines a patient‐specific correction factor by taking the ratio of measured dose and the ArcCHECK planned dose for each voxel to the TPS patient dose for that same voxel, which has its limitations. We also saw bigger differences in point doses, which are attributable to the binning effect and the applied correction factor.

We saw differences at the field edges, which are due to jaw and MLC transmission errors, implying differences in beam models in these two software tools. We could not explore any plans with couch kicks, as the current version of 3DVH does not support such plans. In addition, the current library does not support any 15 MV plans, either, as the 15 MV ACPDP algorithms currently does not exist in the library.

Overall, the real‐time gantry angle comparisons between machine log file and 3DVH agree well. The differences are mostly coming from the temporal resolution for sampling leaf trajectories in the log files and the accuracy of the virtual inclinometer with the 3DVH software. The temporal resolution for sampling machine characteristics on Elekta linacs is 4Hz (or 0.25 seconds), compared to instantaneous readout on the ArcCHECK device.^(8)^ In addition, 3DVH has an inherent inclinometer where the real‐time gantry angle is calculated by the entrance and exit photons hitting the phantom. A slight error can be expected in the inherent inclinometer calculation if the entering photons are scattered at wider angles and do not hit the exit detectors. In the existing study, we could only compare gantry angles between 3DVH file and machine log file. If the machine log files or DynaLog files (Varian Medical Systems, Palo Alto, CA) could be read directly in 3DVH, we can also compare the fluences between the two methods. In that situation, the differences in the predicted delivered dose will solely be due to dose calculation algorithm.

In this study, we used machine log files to reconstruct the dose. As reported in our previous publication, systematic errors in machine parameters can be detected overtime. Our study showed that the mean MLC error is a good indicator of MLC gain calibration. If the MLC gain calibration on the machine is off, the mean MLC error will start to deviate from 0 mm, the direction of which will indicate whether the gain is larger or smaller than the correct value.^(8)^ However, as mentioned previously, a considerable machine QA must compliment log file analyses. The log files, in combination with the postcorrection CBCT of the day, can represent the dose delivered in the anatomy of the day, along with the setup errors. 3DVH can be calculated on the CBCT, as well, but requires a separate set of measurement and will not represent the actual delivery error. Machine log files, in combination with a thoroughly benchmarked Monte Carlo dose calculation engine, may validate the accuracy of dose reconstruction method in a heterogeneous geometry. In addition, they can also be used to investigate and quantify interplay effect between tumor motion and MLC leaf motions. Various studies have looked into the interplay effect.^(14)^ We have also modified the existing design of ArcCHECK for dynamic QA.^(6)^ ArcCHECK absolute dose analysis between measurement and calculation using gamma analysis (3%/3 mm) showed more than 98% of diodes passing for both static and dynamic phantom, with and without the lung phantom insert. MOSFET static measurement showed 2% agreement with the calculated value (5450±120 vs.5250±20 cGy). The dynamic measurement showed a larger spread than calculated (6900±140 vs.7000±250 cGy), indicating an accentuated interplay effect between MLC motion and tumor motion that can also be measured. The novelty of such a modification is that it can be used to perform patient specific 3D QA in heterogeneous lung geometries, taking into account tumor motion trajectories from 4D CT scans.

## V. CONCLUSIONS

In this study we have compared two independent dose reconstruction methods for VMAT QA. Both methods are capable of taking into account the measurement and delivery parameter discrepancy, and display the actual delivered dose in real patient geometry rather than the phantom geometry. The dose discrepancy can be evaluated in terms of DVH of the structures, and provides a more intuitive understanding of the dosimetric impact of the delivery errors on the target and normal structure dose.

## ACKNOWLEDGMENTS

The authors thank the anonymous reviewers for their comments and suggestions.
